# Promoting Awareness about Psychological Consequences of Living in a Community Oppressed by the Mafia: A Group-Analytic Intervention

**DOI:** 10.3389/fpsyg.2017.01631

**Published:** 2017-09-20

**Authors:** Cecilia Giordano, Giusy Cannizzaro, Crispino Tosto, Laura Pavia, Maria Di Blasi

**Affiliations:** Department of Psychological and Educational Sciences, University of Palermo Palermo, Italy

**Keywords:** Mafia, Antimafia, social unconscious, group analysis, group process

## Abstract

The effects of the Mafia have been extensively studied from sociological, economic, and historical points of view. However, little research has investigated the influence of the Mafia on individuals and communities in terms of its psychological and social impact. In order to contribute to the advancement of our understanding of the psychological effects of the Mafia on individuals and communities and to promote a participative process of social change, a group analytic intervention was conducted within a Community Based Participatory Research carried out in Corleone, a small Sicilian town with a historically recognized role in the evolution of the Mafia, as well as in the fight against its control. Qualitative findings from the group intervention revealed the development of an awareness process that allowed participants to become aware of their social unconscious anxieties and defenses and to recognize and manage the strong emotional impact related to the Mafia's presence in their lives. Highlighting how psychological processes can have negative impacts on individual and collective capacity to pursuit transformation and resilience, this article provides important insight on how clinical psychology may operate in socio-cultural contexts to promote the reconstruction of the traumatic social dimensions in the community.

## Introduction

The Mafia is a criminal organization that uses the strength of intimidation to commit crimes and to acquire the management and control of economic activities, public contracts, and services, in order to enhance the control of territory and economic profits (Paoli, 2003; Finckenauer, 2005; Lavorgna and Sergi, 2014). The Mafia's presence has been negatively affecting social and economic development in Italy from more than 150 years (Gambetta, 2000; Daniele and Marani, 2011; Dalla Chiesa, 2014). Specifically, by means of violent and illegal instruments, the Mafia hinders Italian financial, cultural, and social development through its members' infiltration in the most important institutional and political networks (Ruggiero, 2010; Pinotti, 2015; Savona, 2015).

Similarly to other criminal organizations, the Mafia shares in particular with gangs some social and cultural features as the achievement of a higher status, power, respect and a sense of protection from social threats (Taylor et al., 2008; Goldman et al., 2014; Travaglino et al., 2014). With regard to southern Italian countries, criminal organizations provide a distinctive social identity and a sense of belonging that may be particularly appealing to youth that confront high levels of job insecurity, unemployment, and weak youth policies (Travaglino et al., 2014; Di Blasi et al., 2016). Nevertheless, the Sicilian Mafia shows some distinctive characteristics such as a more formal interne structure, well-defined economic activities and political relationships, specific codes of conduct, especially when compared to gangs (Paoli, 2003; Travaglino et al., 2014). Moreover, the Mafia maintains the monopoly of violence and the control of a territory since it illegally ensures several services that the State is unable to provide to citizens (Gambetta, 1993; Paoli, 2003, 2004; Schneider and Schneider, 2005). It should also be considered that the Mafia requires swearing a lifelong allegiance to its membership (Paoli, 2003; Lo Verso and Lo Coco, 2004). Violence is used to ensure the absolute obedience of the affiliates and to punish those who betray or disrespect the rules and the authority of the group. The menace or the effective use of violence is also employed to impose obedience on the communities in which Mafia exerts its influence and to frighten or eventually eliminate those who oppose the power and the economic activities of the criminal organization (Paoli, 2003; Travaglino et al., 2014).

Through an extensive infiltration of the vital fabric of the communities, the Mafia exerts a form of social control that can be described as a “private security system” that ensures protection, while imposing the respect of the criminal organization's rules (Gambetta, 1993). This social control implies submission and *omertà* (the law of silence) for both affiliates and citizens (Paoli, 2003; Lo Verso and Lo Coco, 2004). *Omertà* requires the duty of loyalty, obedience, and silence of affiliates and when these rules are transgressed members are punished through the use of violent and cruel acts (Paoli, 2003). However, the law of silence applies to everyone else and implies to avoid the collaboration with the forces of law and order if being victims or witnesses of illegal activities (Lo Verso and Lo Coco, 2004).

Travaglino et al. (2014, 2015, 2016, 2017), in their recent studies, clearly highlighted how in the Italian criminal organizations *omertà* is a key socio-cultural dimension linked to the concepts of honor and masculinity. The link between honor and masculinity is evident in linguistic expressions such as “men of honor” (Paoli, 2003), which describe the affiliates to the Mafia. A “man of honor” is required to be able to avenge crimes and offenses without the help of the authorities and police. Specifically, individuals' higher acceptance of *omertà* and honor-related ideology was related to both the perception of greater legitimacy of the criminal organizations and fewer collective action intentions against them.

As highlighted in recent literature (Natale et al., 2013; Di Blasi et al., 2015; Nardin et al., 2016; Travaglino et al., 2017), the Mafia affects the Italian context not only from the economic and political points of view but it also weakens the whole community system compromising social capital, values, social identities, and mental representations. This study aimed to deepen these aspects and to show the need for a psychological intervention in social contexts in order to weaken the negative impact of the Mafia on individuals and communities. While the effects of the Italian organized crime have been widely examined from sociological, economic, and historical points of views, little research has focused on the influence that the Mafia exerts on individuals and communities in terms of its psychological and social impact. Over the past two decades, our research team has been studying the Mafia from a psychological perspective in order to achieve a deeper understanding of the culture and mentality underlying the Mafia behaviors from interviews with relatives and affiliates and clinical reports of Mafia members' psychotherapies. These studies have highlighted how the Mafia generates suffering and distress both in its family's members and in its victims and how the identity of Mafia members is inextricably nested in the culture of the families and the community to which they belong (Lo Verso and Lo Coco, 2004; Giordano and Di Blasi, 2012; Giordano and Lo Verso, 2015; Mannino et al., 2015). Moreover, these studies suggest that the identification with the group represents a core dimension in order to explain affiliates' and citizens' attitudes, behaviors, and the overall Mafia's culture (Lo Verso and Lo Coco, 2004; Schimmenti et al., 2014; Mannino et al., 2015). Finally, prevalent denial defense mechanisms to cope with emotions, inflated self-representations, and the lack of guilt observed in Mafia members suggest the presence of specific psychological traits (Lo Verso and Lo Coco, 2004; Schimmenti et al., 2014; Giordano and Lo Verso, 2015).

Recent literature has also provided additional psychological and sociological contributions that examine processes of social change in opposition to the Mafia's control promoted by activists, citizens, and associations. Specifically, the anti-racketeering campaign promoted by Addiopizzo's in Palermo from 2004 represents a significant attempt to carry out long-lasting cultural and social changes involving the civil society and the public administration. Addiopizzo in a unique experience of a bottom-up Antimafia strategy aimed to promote extortion resistance through entrepreneurs' public refusal to pay the racket to the local Mafia and encouraging consumers to practice a critical consumption buying good from firms that joint Addiopizzo. Recently, cultural and social determinants of firms' decision to join Addiopizzo have been investigated (Vaccaro, 2012; Giannone and Ferraro, 2015; Vaccaro and Palazzo, 2015), in parallel with other studies that explored values that can prompt entrepreneurs and consumers to engage in anti-racketeering actions (Natale et al., 2013; Gunnarson, 2015; Elsenbroich, 2016; Marin and Russo, 2016). Nonetheless, little has been written about the Mafia from a psychological perspective taking into account the individual and collective consequences of living in a community oppressed by the presence of a criminal organization.

This article would address this paucity of research showing how the concepts of social unconscious and group processes elaborated by group-analysis may help to understand how social constraints imposed by the Mafia's control over the community impinge on the individual psychological functioning. The group-analytic perspective focuses on the interrelations between social, interpersonal and the intrapsychic levels of individual life experience (Foulkes, 1948). Recently, the concept of the social unconscious has increased in importance in the group-analytic literature. Although Foulkes (1964) mentioned this concept to describe the social and communicative forces affecting interpersonal and transpersonal processes, he did not elaborate further on this concept. The social unconscious has been subsequently defined as a dimension inherent to the social level and structured by social power relations: it is co-constructed by members of the same society or culture and contains the shared unconscious fantasies, myths, anxieties, defenses, and memories of a specific community (Dalal, 2001; Weinberg, 2007). Hopper provided a detailed definition of this concept: “The concept of the social unconscious refers to the existence and constraints of social, cultural, and communicational arrangements of which people are unaware. Unaware, in so far as these arrangements are not perceived (not “known”), and if perceived not acknowledged (“denied”), and if acknowledged, not taken as problematic (“given”), and if taken as problematic, not considered with an optimal degree of detachment and objectivity” (2001, p. 10). Additionally, Brown (2001) stated that the most powerful defenses to avoid pains or deep conflicts linked to social traumatic experiences or painful historical events are denial, projection, and the repression of memory.

Recent research developments have focused on the analysis of the social unconscious in relation to social trauma and transgenerational enactments, as in the case of the Holocaustus (Wilke, 2016), the Palestinian and Israeli conflict (Doron, 2014; Even-Tzur, 2016), and to social inequalities linked to cultural hierarchies of sexism, racism, classism (Layton, 2007; Geyer, 2017).

These studies addressed the question of how enactments of the social unconscious can be studied on a wider social level in clinical and non-clinical settings and showed how trauma or social, cultural, and political events stay alive in the collective consciousness of groups and communities by means of shared stories and narratives. In line with these studies, we hypothesized that the presence of the Mafia in the vital fabric of a community, as a permanent painful dimension affecting social power relations, requires specific social, cultural, and communicational arrangements of which people are unaware, or that are not known or denied. The obedience to the law of silence (*omertà*), strengthened by the Mafia through the massive use of violence and intimidation, contribute to maintain not only the *status quo* but also to the enactments of social unconscious elements that weaken citizens' capacities and intentions to oppose the Mafia. Adopting this point of view may help recognize psychological, cultural, and social dimensions that may increase individual and collective support for the status quo (Travaglino et al., 2017), and overcome reductive perspectives according to which Sicilian people display a collective passivity in opposing the Mafia only due to legitimization processes (Sciarrone and Storti, 2014) or to widespread forms of social consensus linked to ethical bases such as amoral familism (Banfield, 1958).

According to Foulkes (1948, 1964), each individual is inevitably determined by the world in which he lives, by the community, the group of which he forms a part and common social perceptions and experience linked to this belonging penetrate the inmost being of the individual personality.

As within the group analytic approach the group allows the analysis of both the individual and social unconscious, it is a very appropriate instrument to help people develop an understanding of their psychic and social experience. Providing a theoretical and clinical container to elaborate thoughts and feelings (Foulkes, 1964), the group experience promotes a change by creating a holding environment for analyzing, understanding, and becoming aware of the social unconscious dimensions in the group and in society (Weinberg, 2007).

The present study reports a group analytic intervention aimed to enhance participants' awareness through a shared reflective process about the social unconscious dimensions, in terms of affects and defenses, linked to the presence of the criminal organization in the territory. The intervention took place within a Community Based Participatory Research (CBPR) conducted in Corleone, Sicily (Italy), a territory with a significant historical role both in the evolution of the Sicilian Mafia and in the fight against Mafia organization. Specifically, during the years 1970-1980, the clan of Corleonesi, headed by Luciano Liggio and Salvatore Riina, became dominant within Cosa Nostra. In the early 1980s, Riina and Provenzano decimated other Mafia clans through a fierce mafia war that caused hundreds of murders including heads of rival clans and members of Antimafia (Andretta, 2005; Lupo, 2010). Many of these victims were children and women who were often murdered by horrifying methods, such as being liquefied into acidic compounds (Schimmenti et al., 2014). On the other hand, the Anti-mafia movement in Corleone has an important tradition that since the nineteenth century began with trade union activists and cooperatives of agrarian workers (Santino, 2009; Rakopoulos, 2017; Scalia, 2017). With this regard, the story of Placido Rizzotto, a union trade activist killed by the Mafia in 1948, is emblematic since his mutilated body was found only in 2009. The figure of Rizzotto has been evoked in numerous films and television series and a red wine produced by the Antimafia cooperative “Libera Terra” still bears his name. Moreover, the first confiscated good, belonged to Riina the leader of the Sicilian Mafia, was a vineyard located in Corleone. In 1999, the plot was conferred to the “Lavoro e Altro” cooperative. As Rakopoulos (2017) underlined, this was the beginning of the formation of an Antimafia cooperativism system in Corleone territory that today involves about 10 agrarian cooperatives across Italy. As mentioned above, in order to understand the relationship between Corleone citizens and the Mafia, it is important to consider that social and historical trauma or/and cultural and political events can persist in the collective consciousness of groups and communities producing a damning impact in terms of individual and collective psychological functioning (Mohatt et al., 2014; Geyer, 2017). Moreover, to avoid conflicts and anxieties linked to these social traumatic events, powerful defenses as denial, projection, and the repression of memory are activated at a social unconscious level (Brown, 2001). These unconscious psychological arrangements are reinforced by some cultural, political and anthropological aspects such as *omertà* and a widespread distrust in institutions and law enforcement, too often ineffective or colluding with the criminal organization. The interplay between psychological, socio-cultural, and political elements affects the capacity of Corleone, and overall Sicilian, citizens to successfully oppose to the Mafia's control over their lives.

## Methods

The present study sought to analyze a group-analytic intervention carried out in order to promote participants' awareness of the adverse effects of the criminal organization on their lives. Group meetings took place within a CBPR conducted in Corleone, Sicily. The CBPR started in January 2011 and ended up in January 2012 and was developed through three interrelated steps: the context analysis, the group meetings, and the final public meeting. The partnership was composed by the academic team (two senior researchers, who were also the conductors of the groups, and two facilitators), the Municipality of Corleone, and a group of community members (local administrators, teachers, college students, housewives, and representatives of Antimafia associations).

### The context

Since the Mafia spreads differently depending on socio-cultural features that characterize a specific geographical area, a strong focus was primarily maintained on the request for the intervention and on the link between such a request and the current features of the local community. Specifically, the request for the research was introduced to the team by a psychology student at the University of Palermo who had previously met the City Mayor. Following the request reported by the student, the team met the mayor, and other local administrators individually in order to clearly define the motivations underlying the request for the intervention.

As mentioned above, the Mafia has affected the history of Corleone and its citizens' social imagery, and some relevant events had occurred at the time when the intervention was requested. Some months earlier, one of the mafia boss Salvatore Riina's sons returned to Corleone to undergo preventive special surveillance, after he had been imprisoned for 8 years and 10 months for Mafia association crime. This event upset Corleone's inhabitants: once again they were forced to share their everyday living spaces with a Mafia bosses' family members and experienced worries about the risk of overbearing behaviors and threats to individual freedom and safety. In fact, only a few bosses' family members have chosen to live outside Corleone during their relative's hiding or imprisonment. Moreover, many bosses' siblings, children, grandchildren, wives, and sisters-in-law currently live in Corleone. As a consequence, Corleone's citizens often meet Riina's wife or Provenzano's children during their daily commitments. Acting on behalf of most Corleone citizens, the mayor took a very courageous stance. He declared to the press: “Riina's junior is not welcome here.” First, such a strong public declaration produced the mayor's awareness that he was not opposing only an individual (Riina's junior) but a criminal organization as a whole. With regard to citizens, they had to take a stance: either with the mayor or with the Mafia. Most of the citizens had no doubt over which side to choose and felt represented by the mayor's words, however, it is likely that some felt unable to decide because the conflict was too strong. Furthermore, for a small minority, the mayor's statement was probably interpreted as an offense to the criminal organization.

Five meetings with the mayor and local administrators allowed the team to better understand the meaning of the intervention request: on one hand, the mayor and municipality, at a sensitive time in the town's history, wished to strengthen their protection network by giving voice to many honest Corleone citizens' consensus; on the other, they wished to provide citizens with a space where they could elaborate feelings prompted by the presence of the boss's son in town. Drawing on these lines, after the meetings, the following goals were planned: (a) helping Corleone citizens to share emotions and fears linked to their cohabitation with the Mafia; (b) promoting an emancipation process from Mafia oppression, starting from an increased awareness of the thoughts, behaviors, and feelings that contact with the Mafia arouses in people; and (c) analyzing the psychological and social consequences produced by organized crime in the local population and detecting consensual future developments.

Moreover, the psychology student, who acted as a facilitator, was in charge of inviting people to participate in the CBPR introductory meeting during which the team announced that it would be possible to register for discussion group meetings so as to actively participate in the CBPR. Given that some people might need more time to decide to actively intervene when a relevant them such as the Mafia is dealt with, researchers added that people could attend the first group meeting even though they had not previously enrolled.

### Group meetings

The CBPR approach conceptualizes community as a dimension of collective and individual identity in which individuals share common symbolic systems, social norms, interests, and needs(Israel et al., 1998; Wallerstein and Duran, 2008). Consistent with this theoretical framework, the group analytic approach focuses on the interrelations between individuals, families, community, and social network (Foulkes, 1948). Specifically, group analysis is based on the principle that human beings are deeply social by nature since their lives are inextricably linked in many ways. Given that group membership reflects the wider norms and values of society, the group is a privileged tool to explore social unconscious dimensions and promote deep lasting attitude changes by facilitating interaction and for exploring social unconscious (Dalal, 2001, 2011). Consistent with other participatory research studies utilizing psychodynamic groups in educational and work settings (Leclerc and Maranda, 2002; Newton and Goodman, 2009; Giannone et al., 2015), the group work promotes participants' interactions, aiming at an intersubjective analysis of their experiences and feelings. The conductor's capacity for holding is crucial to creating a safe communicative space (Newton and Goodman, 2009) in which feeling and emotions can help participants in collectively reconsidering their relations to each other and to their environment.

As previously pointed out, one of the biggest difficulties in the fight against the Mafia is *omertà*, defined as an implicit law of silence that originates from a fear of retaliation, and conveys a direct or indirect solidarity with the authors of a crime. As a consequence, participating in a group where publicly talking about the Mafia is required means, first of all, breaking the law of *omertà*. An individual who expresses his/her point of view against the Mafia is, at the same time, engaged in an action of strong political, social, and individual significance, thus experiencing deep unpleasant emotions. For this reason, it is particularly important to build a relationship based on trust with the partnership, as highlighted in the literature (Christopher et al., 2008; Shalowitz et al., 2009; Lucero et al., 2016).

In this study, the group meetings took place at the Mafia boss Bernardo Provenzano's house, a seized asset and the seat of some cultural associations committed to the fight against the Mafia, which promotes awareness campaigns and training courses. Currently named the Legality Lab, it is located close to the house where Provenzano's brother still lives. This location contributed to making the experience emotionally intense due to repeated intimidating acts which have been occurring over the last 20 years in Sicily and Corleone against the managers and users of seized and confiscated assets.

The team expected a numerically poor participation to the group. During the introductory meeting, only 14 citizens had registered to participate in group meetings. Despite being the first time that research against the Mafia involving citizens' active participation had been proposed in Corleone, unexpectedly, 31 citizens attended the first group meeting. Since the number of participants was greater than fifteen, the research team decided to set up two groups, each with a conductor and a facilitator. Only one of the two groups was entirely recorded and transcribed after consent from all participants was obtained, while the second group could not be registered because two participants did not give their consent. In the latter group, the facilitator acted as a recorder, transcribing the most significant sentences and aspects of the process. The group's work included three meetings of two sessions, lasting for an uninterrupted 75 min with one break of 20 min, using an intensive group format over 3 days. Two senior researchers, who are also group analytic therapists, acted as conductors.

The first group consisted of 16 participants (9 female), aged between 18 and 62, while the second group consisted of 15 participants (7 female), aged between 19 and 58. Two group-analytic therapists lead the groups, encouraging the establishment of a free-flowing discussion. The group analytic therapist tends to be referred to as a “conductor” rather than a “leader” (Foulkes, 1948). This reflects both a strong democratic principle that underlies the group analytic approach, and an understanding that the therapist's role is not to start a discussion or overly direct the group, but to interact in order to help the group make sense of the stories that are told, the feelings that resonate, and the resonances and dissonances that arise.

## Results and discussion

The present study reports the experience of a group analytic intervention aimed to enhance people's awareness through a shared reflective process about the social unconscious dimensions linked to the presence of the criminal organization in the community of Corleone. In this section, we sought to highlight some significant passages characterizing the group process that led participants to recognize and share unconscious or unknown feelings and emotions linked to the influences of the criminal organization on their lives. During the early phase of the first group meeting, discussion of the Mafia took place at a shallow level, highlighting both the need for putting aside an early personal and emotional involvement and the presence of denial defenses to avoid pains or deep conflicts linked to the social unconscious dimensions related to the Mafia (Brown, 2001; Hopper, 2001). Thereafter, starting from some interventions about personal daily experiences, the law of *omertà* was broken, thus allowing most of the members to express doubts and difficulties in sharing everyday spaces with bosses and their relatives. These shared narratives represented the first important passage allowing participants to voice the “unthought known” (Bollas, 1989) about the Mafia and showed the presence of specific social and cultural constraints of which participants were unaware before (Hopper, 2001). The possibility to consciously think about their past and current experiences allowed participants to make a further progress during the second meeting, coming into contact with painful feelings and emotions, and making them available for thought. Through this second passage, participants could start to recall traumatic memories and events and recognize that they had built strong defenses and developed specific behaviors to avoid the sufferance the Mafia had caused in their lives (Weinberg, 2007). During the third group meeting, the previous emotive and cognitive engagement helped participants to recall and re-interpret their current and past experiences, increasing awareness of the limitations of freedom and expression that the Mafia imposes on their lives and their community. Becoming aware of this influence and sharing ideas about the modalities available for coping with it was the third and final step of the group process.

### First group meeting: voicing the “unthought known”

At the beginning of the first group session, the conductor asked the participants to introduce themselves and explicate their expectations with respect to the experience they were about to start. Everybody initially assumed a “passive” position with respect to the group experience. Many participants seemed to be pushed by curiosity and intellectual interest, rather than the need to share lived experiences and thoughts connected to the Mafia. Moreover, as the following quotes show, great diffidence and skepticism initially seemed to linger in the group, and many participants expressed a stronger need to listen rather than intervene.

My name's Laura, I'm an office worker at school. The topic we are talking about is interesting; however, I'm not quite sure things will change. Sometimes it seems that everything is going to change but eventually it remains the same…In short, I count on young people…

My name's Mario, today I'm here as a listener, may I?

My name's Piero, I'm an estate agent and curious to listen to all of you.

I'm a police commissioner. I'm here because I've been invited. I'm not here to talk because in that case I should be authorized by the police Headquarters…

However, some young participants started to show a greater propensity to confront and share their experience from the beginning.

*My name's Paola and I'm from Corleone. Going into this theme is such a great pleasure for me because I'd like to have a confirmation about the stories my parents have been telling me. I'm pleased to talk about this theme with people of my age, as well as with all the people present here*.

*My name's Maria, I've got a degree in Educational Science and I'm working in Palermo. I chose to participate because I consider this topic very interesting and also wish to talk about my experience*.

Thereafter, some participants started to discuss the Mafia through a narration of their personal experiences, specifically talking about the “law of silence.” They spoke about the trouble of living in a city where they often feel observed not only by the Mafia but also by law enforcement agencies and others citizens. They also reported episodes that highlight the anxieties and difficulties caused by sharing daily life spaces with Mafia members' families, as the quotes of three participants below illustrate.

*I wish to talk about my everyday life in Corleone. As well as being an employee, I also work here as a municipal councilor. My father is a retired police officer and, even though my parents are not from Corleone, I was born and grown up here and I hope to continue to live in Corleone (…) Since I have been living in this town, I know who belongs to the Mafia and who doesn't and I also know how to behave, even though it's not always easy… (…) Every morning I go to a café to have breakfast; sometimes it can happen that some people connected to Mafia families enter that café so I have to choose, in a heartbeat, if I have to say hello to them or not. Sometimes I realize that some policemen are coming in at the same time. I feel cornered, at a turning point, and wonder: What if I don't say hello, close my eyes, play dumb and go away? Should I say hello even if I know that the policeman could write in a report “Today the municipal councilor said hello to the dude at the café”? Almost every morning I usually find myself at this turning point*.

*My fiancée is not from Corleone, so she doesn't know the houses where Mafia family members live. Sometimes she points her finger at a house and says: “Look how beautiful that house is!”. Aware of the fact that Mafia members are living in that house, I beg her to lower her finger. (…) It's great to talk about the Mafia but it's really difficult to talk about the Mafia in Corleone*.

*Let me tell you one of my experiences…When I was at school, one day a teacher told us: “Guys, let me inform you that in our class there's going to be a new student. I would highly recommend you have to behave properly and, starting today, we will not talk about issues relating to the Mafia”. My new classmate was the daughter of a boss. My classmates and I were shocked, we were afraid. However, we started a more or less friendly relationship with her, despite the fact that we knew that we had to be careful what we could or could not say when we were talking to her*.

During the first group meeting, a lot of questions, doubts, and dilemmas emerged. Moreover, the quietest participants nodded while hearing other people's tales of their experiences. It seemed clear that all participants had found themselves at a challenger turning point at least once in their life: “Should or shouldn't I say hello to the boss's relative? Should I welcome him into my house? How can I conquer my fear of talking about the Mafia if, since I was a child, adults have always taught me not to speak about or oppose to the presence of the Mafia in my town?”. Painful conflicts not clearly perceived before that time or not “known” (Hopper, 2001) linked to the experience of sharing spaces of everyday life with Mafia members' children and relatives started to emerge and became an important theme during this meeting. In this first meeting, the main conductor's tasks were to create a safe communicative space, a holding environment in which both cognitive and emotional unconscious or unknown feelings in relation to the Mafia could be listened to. Such a holding environment supported the emergent understanding of an important passage: from the narration of the Mafia as an external criminal organization far from citizens' lives to the narration of a present and close Mafia who observe, impose silence, and influence community members' public and private behaviors and lives. To summarize, the group process started to work for participants as a holding environment in which they could analyze, understand and become aware of the social unconscious processes they shared as a result of living in a close contact with the Mafia.

### Second group meeting: voicing emotions and feelings

The second group meeting was characterized by the possibility of sharing emotions mainly connected to past events of Corleone's history within the group. The holding environment built in the first meeting allowed participants to reveal, and later elaborate on, the painful emotions that they had experienced in relation to the Mafia. As Weinberg (2007) pointed out, the group process allows members to unconsciously re-live and re-enact in the here-and-now relationships emotions related to painful or traumatic past events. The role of the conductor was mainly oriented to facilitate reflective and constructive encounters with the past.

The word “fear” was often present in several interventions. For example, a mother talks about the fear for her children's safety that she had been feeling for many years:

*I felt scared when my children, when they were kids, lingered on the street with friends. Sometimes they could have been talking with a boss's son…and I was afraid they could be involved in an ambush…even if you were not the target, you could be killed…It could have been fatal!”*.

In another example, a young woman remembers her frustration during adolescence, presenting the following episode:

“*Once, while I was joking with my friends, I pronounced the name of a boss…They glared at me and looked around scared to death…From that day on, I understood that I had to give up my spontaneity, the spontaneity of my language*”

During this second group meeting, some participants could also tell the group about memories connected to Mafia violence.

I remember that there was a local festival and many people around. Suddenly, we heard a woman yelling “Bastards, bastards”. My sister and I got closer to the place these voices were coming from. A woman was shouting “They killed him”. We stopped in front to the place of the homicide, I saw a white car—I'll never forget—and as soon as my sister understood, she immediately pushed me in such a way that I couldn't see anything!

…*Years ago there was a murder and my father, who was working at the Town Hall (…), was called very early in the morning because he was asked to clean the roads. When he came back, he told us about this river of blood which was flowing down…I was still too young to understand these things, but I could see my mother's fear every time my father had to go and pick some panjandrums at the airport or take the mayor somewhere…I really remember this fear…*

These narratives opened a space of resonance and mirroring (Foulkes, 1948) in the communication process of the group thus activating a growing emotional flow. Participants could share emotions and anxieties connected to the Mafia and recognize the relevant psychological and social effects that organized crime had caused in local people.

Almost at the end of the second meeting, a young woman's intervention moved the group members, who could finally recognize the suffering the Mafia produces in citizens and the community.

For me, the Mafia is pain, an immense dark pain. The pain I feel is not abstract… (…) one of my schoolmates suddenly disappeared: he was first killed and then chewed up by dogs. Then they put him in a jeep and set it on fire. (…) From that moment onwards I escaped and stayed away from Corleone for many years; I didn't want to live there any more… (…)

*Mafia is pain, a strong pain which is also present in Mafia relatives. (…) Sometimes, when I speak with these people, I realize the immense dark pain they feel inside; they'd like to talk to you, but cannot; they do not reveal much, but they have passed down a huge pain*.

During the second group meeting, the holding environment enabled participants to recognize their shared painful emotions linked to the violence of Mafia, which they had had to keep silent for a long time. In a recent review on the impact of historical trauma, Mohatt et al. (2014) proposed a perspective that interprets trauma as a psychological process independent from the specific traumatic event, as a narrative representation of the past that contains both personal and collective components and that continuously affect present-day representations of people. Through the possibility of sharing public narratives about violent and traumatic social unconscious dimensions participants could recognize the psychological suffering the Mafia had produced over the years, as well as the pain rising from the awareness of feeling oppressed by a hidden power that was, at the same time, clearly visible in many inhabitants' faces and stories.

Finally, it is important to note that at least two elements contributed to promote the willingness to explore these social unconscious dimensions in the group. First, the capacity of the conductor to encourage and contain the emotions that surfaced. Second, the emotional group dynamics are also influenced by the affective dimension of group identities and by people's specific interest in a particular situation (Hoggett and Thompson, 2002). As a consequence, the affective and current factual dimension of the Mafia in the group culture of Corleone's citizens made them passionate and willing enough to express their feelings in a safe communicative space.

### Third group meeting: from emotions to awareness

Sharing unconscious emotions and aspects connected to the Mafia allowed participants to gradually become aware of the power that the Mafia has exerted over them and to reflect on personal and collective initiatives to cope with the threat of the Mafia in everyday life. Two participants stated:

*I paradoxically came here to meet the Mafia through your stories (…). After these group meetings, I realized I had seen, met the Mafia, but I didn't think that the Mafia also meant this…I met it, let's say, in many small things, in many little moments of my life (…) I realized, for example, that the family who was living next door when I was on holiday was a Mafia family*.

*Once, I was in the garage with my boyfriend (…) he comes from a different town (…) he is more free from such concerns (…) a child, son of the Boss living in my apartment building, deliberately stepped on my boyfriend's foot with his bicycle. My boyfriend gave him a slap and I've been scared for a week (…). I realize now how much I have been conditioned*.

The quotes mentioned above clearly show how the frequent contact with Mafia's member or relatives in everyday life caused anxieties and pains against which participants had built strong defenses in order to protect themselves. As highlighted by Weinberg: “Uncovering these traumatic memories or the way they unconsciously impact on a society is the essence of the Social Unconscious.” (2007, p. 316). Mutual resonance and mirroring facilitated in the group the emergence of analogous narratives increasing participants' awareness growth.

Subsequently, a participant shared with the group a narrative about his parents' ways of coping with the perceived threat exerted by the Mafia that provides an example of the reenactment of the memory of a suffered humiliation:

My father is a customs officer and his station has recently been transferred near to Totò Riina's house (…) Once, he told me he had to leave his station with his service car but he found another car closing the exit (…) he began to honk and, after a really long time, Riina's wife and their daughter (…) she moved her car apologizing (…) my father is a big man and he was driving his service car (…) Do you think that he was able to affirm the government power? (…) but would he be able to do it as an ordinary man?

During the third group meeting, participants could also recognize that the Mafia strongly limits their personal freedom. For example, noting this negative influence, two participants said:

I live in a special apartment building where Mafia families also live. Every day I have to deal with people who literally limit my freedom!

*I lived in Palermo to attend the university (…) I come from a town in which the presence of the Mafia is relevant and nothing shocking has ever happened to me. However, I've reached a deeper understanding of many events of my life in Sicily by participating in this group (…). My father used to bring me to Palermo with his car and to park it in a small street near my house (…). He always found a flat tire or a rear-view mirror (…) It did not happen by accident, we could not park the car in that street (…) A girl living in my building also found her car to be burned twice*.

The above reported quotes represent a shared anxiety among Corleone's citizens, and they mirror the original motivation for the intervention request. The return of the Boss' son to Corleone seems to have created an emotional imbalance in its citizens. His homecoming compelled them to face the painful experience of living once more with the frustration of being silent, obeying the Mafia codes, and being limited in one's own freedom of expression and action.

At the end of the third group meeting, the conductor pointed out some aspects which highlighted the possibility of giving a shared meaning to social unconscious emotions felt outside and inside the group.

*One might think that the idea that the son of the boss came back and we do not want his presence here is, like,…an ideological or political opinions…In contrast, in the course of these meetings, you have spoken of a strong emotional imbalance. As though Corleone had suddenly lost a bit of serenity and integrity laboriously acquired before. The story told by one of the participants can be considered a strong metaphor of this process. Just like Maria's father, Corleone had to clean its roads from the blood that the Mafia had spilled! Therefore, the idea that the boss's son is returning causes an imbalance, makes you feel the smell of blood once more. In my opinion, during these group meeting, a mostly emotional process took place which had its peak, when you spoke about your pain, the deep pain that Corleone has been experiencing and that strongly touched us, mostly because it's as if you had spoken about the most brutal aspect of the Mafia, that is its inhumanity, the annihilation of freedom which had corroded the community's social networks*.

During this last meeting, the experience of a safe communicative space allowed the expression of the unconscious and unthinkable about the Mafia and made it possible for participants to contemplate new thoughts and hope for a less problematic future. Below, a participant's intervention explains his perspective on the group meeting experience:

*I had the feeling that we did something which in Corleone had never been carried out (…) because each one in the group has given evidence of his or her experience or anyway told his or her personal opinion and had the chance to confront with other citizens about the Mafia. (…) For those who were present, this has been something very strong which has left a seed. I'm sure that this work is really useful, way more than any face-to-face meeting, demonstration, book presentation, because I had the feeling that we were willingly putting ourselves in the first person and this allowed us to make a step up. In my opinion, this has been important for all participants and for our community*.

This last quote highlights how the enactment of social unconscious aspects through public narratives of significant shared painful experiences could sustain resilient responses. As suggested by Mohatt et al. (2014), historical trauma narratives could inhibit psychological growth and collective future-oriented aspiration or sustain lasting community transformation and resilience. One of the most important challenges that clinical psychology has to deal with operating in socio-cultural contexts is to investigate and identify the conditions that could sustain the change and the reconstruction of the social traumatic and conflictual wounds in the community.

## Conclusions

As Geyer (2017) suggested, the group-analytical oriented group provides a window into the social unconscious and the unconscious power relations that sustain it, showing to be a very appropriate tool for research issues analyzing social change processes. In this paper, we sought to broadly report the most salient passages of a group-analytic intervention highlighting how the enactment of social unconscious dimensions allowed participants to become aware of the negative influences exerted on them and on the community of Corleone by the presence of the Mafia. This experience provided to participants a greater ability to manage the negative impact caused by the Mafia in their lives and this could promote a future-oriented capacity to oppose the criminal organization. Indeed, during the group meetings, participants could experience a holding environment in which they could become aware of their social unconscious anxieties and defenses and find new ways for re-thinking and interpreting their cognitive and emotional experiences, as well as discussing further collective actions to oppose the oppression of the Mafia. Although for the first time, this intervention gave participants the chance to be aware of the ways in which the Mafia oppresses their lives, one important limitation needs to be acknowledged. Due to the lack of financial resources, this intervention was time-limited and involved a small number of citizens while a more effective action would have required a more extensive participation and a long-term intervention. Nevertheless, we think that this intervention could represent a useful model to analyze the mutual influences between social phenomena and processes and subjective experience and to inspire future psychological intervention to promote lasting changes in the fight against the Mafia. Because no single approach by itself can fully be effective to promote social change against a complex phenomenon such as the Mafia, this article sought to highlight that psychological processes can have damaging impacts on the capacity of individual and collectivity to pursuit transformation and resilience. This evidence also provides important insight on how clinical psychology may operate in socio-cultural contexts to promote the reconstruction of the traumatic social dimensions in the community.

## Ethics statement

This study was carried out in accordance with the recommendations of the Ethical Code of the University of Palermo and of the Code of Ethics approved by the General Assembly of the Italian Association of Psychology held on March 27, 2015. All subjects gave written informed consent in accordance with the Declaration of Helsinki. The real names of participants have been replaced with pseudonyms to ensure confidentiality and anonymity.

## Author contributions

CG, GC, CT, LP, and MD had substantial contribution to the conception of this work. CG and MD designed the study. GC, CT, and LP collected and analyzed the data and all authors had substantial contribution to the interpretation of the data. CG and MD drafted a previous version of this article and all authors critically revised it for important intellectual input and finally approved of the version to be published. All authors agree to be accountable for all aspects of the work in ensuring that questions related to the accuracy or integrity of any part of the work are appropriately investigated and resolved.

### Conflict of interest statement

The authors declare that the research was conducted in the absence of any commercial or financial relationships that could be construed as a potential conflict of interest.
